# On the Management of Perforations of the Membrana Tympani

**Published:** 1887-05

**Authors:** William B. Dalby

**Affiliations:** F. R. C. S., M. B. (Cantab.) Aural Surgeon to St. George’s Hospital


On the Management of Perforations of the Membrana Tympani.—By Sir William B. Dalby, F. R. C. S., M. B. (*Cantab.*) *Aural Surgeon to St. George's Hospital.*

If any examples of a morbid condition of the body might be described as having individualities of their own, as exhibiting various moods, or showing diversity of behavior under treatment, surely it would be perforations of the tympanic membrane. To those who are in the daily habit of observing how soon they occasionally heal, how for long periods they do not heal (periods to be measured by years, and even by long life-times), how tolerant at one time they are of manipulation and of treatment, how intolerant at others, how susceptible to climate, to diet (especially in the matter of stimulants), they indeed display an "infinite variety."

Occasionally there may be found fragmentary additions to medical literature in which this application or the other is recommended as the best treatment for perforations. Now, although many of these applications are useful under appropriate conditions, I fear it is nearer the truth to say there is no best treatment that may be employed by those who appreciate the interesting variety referred to as belonging to perforations.

To begin with early life. Of all children who during their infancy have a purulent discharge from the ear, after a few hours of pain in the ear (the position of the pain being generally unrecognised at the time), how very small a proportion are submitted to an examination of the membrana tympani! When in isolated cases they are so examined at the time of the discharge, is it not almost the rule that the membrane is found to be perforated? The probabilities, therefore, are that in all, or at least most of them, these discharges are accompanied at the time by perforation. I say at the time, for when the membrane is examined weeks or months after the discharge has ceased, it is often discovered to be entire. We must admit, then, that during infant life the membrane is extremely prone to become perforated, and happily equally prone to heal. This view must have occurred to so many that it is only necessary to mention it, in order that either the occurrence of the perforation or its healing should not excite surprise with those who see comparatively little of ear-disease.

What is still further deserving of notice is that the majority of these children, when they grow up, hear well. If this fortunate ending of the affair is occasionally in part due to the fact that there has been no local interference, it does not follow that nothing in the way of management should be done for recent infantile perforations; but I feel convinced that they are better without treatment, if such treatment includes mineral astringents in solution; the sulphate of zinc and acetate of lead lotions, which used to be thought as necessary for a perforation as iron and quinine mixtures and Latin verses are still for the English boy, are worse than useless. Scrupulous cleanliness—not only by removing discharge from the meatus, but by its expulsion from the tympanic cavity, vegetable astringents, and protection from the action of external air—include the routine of treatment under which the perforations are aided in healing. The same will apply to the treatment of recent perforations in adults, and when the perforation has healed, so long as there is undue secretion within the tympanum, if the case is treated as one of catarrh, the best possible results as to future hearing will follow. Why, then, do we find so many perforations which have occurred from simple catarrhal inflammation either permanent, or, if they heal, leaving such considerable losses in hearing? Of course the disorganization of the tympanic cavity (the result of inflammation)

must be taken into account; but, having excluded this element, I cannot but think this is sometimes due to the pardonable anxiety of the patients to (as they express it) "stop the discharge," and so they resort to anything that offers this prospect.

No doubt, for a time, the mineral astringent will do this, but it does more—it stops the healing, and it stops the hearing. For it astringes the tympanic end of the Eustachian tube; it prevents the possibility of expelling the discharge from the tympanum through the perforation; it allows the discharge to collect in the tympanic cavity, and become inspissated; it causes cicatrization of the edges of the perforation—in short, it does not give the lesion a chance of repair. More than this, when the mineral astringent whose motto is, "Stop the discharge," is discontinued, the discharge comes on as before, so it indifferently fulfils its own motto. I suspect that the advertised lotions and drops for the ear which patients obtain and use on their own responsibility contain mineral astringents in considerable quantities, as they sometimes cause great pain, and excite inflammation as soon as they reach the perforation. If it is thought that the expulsion of the discharge from the tympanum through the perforation tends to keep open the perforation, let it be remembered (and it is a matter of common knowledge) that if a membrane is inclined to heal, nothing of this kind prevents it; of this fact we have ample evidence in the case of perforations made for remedial purposes in cases of chronic proliferous catarrh; and it surely is desirable in the instance of perforation caused by disease that at the time when this happens as little as possible of the products of inflammation should be left within the tympanic cavity.

And now in regard to the tolerance or intolerance respectively which perforations of long standing exhibit to well recognized and useful applications. Take for example the case of alcohol. Many perforations with unhealthy edges, discharging in large quantities from the tympanum, at once become more healthy under the use of alcohol in various dilutions; but to some, alcohol, even extremely diluted, is most painful and irritating. This is especially noticeable if the perforation is of small size. How can it safely be said in a general way that alcohol is a good treatment for perforations? The same may be said of iodoform or boracic acid. What satisfactory results either of these applications often produces in old perforations with bone granulations and a fœtid discharge! But the fact that they occasionally set up irritation is enough to prevent me, and probably many others, from recommending them when the patient is subject to pains in the head, especially if these pains are chiefly on the same side as the perforation.

The tolerance and intolerance of all forms of artificial membrane or support which perforations show is so well recognized that it is hardly deserving of mention; and the excellent hearing and improved local condition of the perforation is perhaps better known than occasional intolerance which some perforations show to its employment; but the following case will perhaps illustrate so well what I might term the behavior of a perforation under treatment that I relate it.

About fifteen years ago, a gentleman had perforations of both membranes after scarlet fever. On the right side all hearing was lost and the perforation healed. It was then found that on the left side (he being extremely deaf with this ear), he could obtain excellent hearing by the use of a cotton wool pad, which he learned to apply to the perforation. This he habitually used during fourteen years spent in active work which necessitated good hearing. Finding one day that the pad did not give the usual result, he tried various applications without avail. Having noticed that the pad was very dry when it was removed, he proceeded, on his own account, to irritate the ear in several ways in order to produce a discharge. Amongst other plans which he tried, one was by dipping the pad into turpentine. This was soon followed (strangely enough, without any pain) by a discharge, and on again adjusting the pad he heard well for several days. Finally, nothing succeeded; the ear became dry and he was deaf.

When I saw him I found, as he told me was the case, that the perforation had healed. The size of the old perforation was markedly shown and filled up with new tissue. After I had made an incision into the membrana, and after a great deal of trouble, the new tissue disappeared, leaving the old perforation as before, and he could again use the pad with efficiency, and without the tendency to heal being shown. Could anything illustrate better than this little history the behavior of perforations? One might almost say that it is necessary to make use of our experience to anticipate their moods, and even then we shall be often wrong in our surmises. What is needed, however, to treat satisfactorily perforations of the membrana tympani, and many other forms of chronic disease, is the intelligent co-operation of the patient, and whoever is the subject of them must patiently learn to manage himself. When it is remembered that by careful management the hearing may frequently be made and kept good enough for the ordinary purposes of life, and, more important still, the patient may be often kept free from the dangers which have a fatal ending, it is not trouble ill-spent.

Of all the many forms of perforation, some of the most troublesome are perforations of Shrapnell's membrane, and the most difficult to treat are those in which there is evidence of caries. The easiest to treat are perhaps those in which the perforation has arrived at a dry state without any discharge, a state which it may retain for many years if not interfered with ; then a masterly inactivity, as shown by leaving it alone (it may be permitted to say for this perforation, if for none other) is the best of all treatments.—*British Medical Journal.*

